# A Sustainable and Antimicrobial Food Packaging Film for Potential Application in Fresh Produce Packaging

**DOI:** 10.3389/fnut.2022.924304

**Published:** 2022-07-07

**Authors:** Ling An, Xinzhong Hu, Phil Perkins, Tian Ren

**Affiliations:** ^1^College of Food Engineering and Nutritional Science, Shaanxi Normal University, Xi'an, China; ^2^Solaster LLC, St. Augustine, FL, United States

**Keywords:** N-halamine, MC, antibacterial, active food packaging, polylactic acid

## Abstract

N-halamines are a group of compounds containing one or more nitrogen-halogen covalent bond(s). This high-energy halide bond provides a strong oxidative state so that it is able to inactivate microorganisms effectively. In this study, a sustainable film was developed based on polylactic acid (PLA) with incorporated N-halamine compound 1-chloro-2,2,5,5-tetramethyl-4-imidazolidinone (MC), as a promising antimicrobial food packaging material. Results showed that the incorporation of MC prevented the crystallization of PLA and improved the physical properties of the films. In addition, both the moisture barrier and the oxygen permeability were improved with the presence of MC. Importantly, the antimicrobial film was able to inactivate inoculated microorganisms by a factor of seven log cycles in as little as 5 min of contact. Films that contained higher levels of MC further enhanced the antimicrobial efficacy. Fresh strawberries packed with the fabricated films maintained the quality for up to 5 days. Due to the ease of fabrication and the effective biocidal property, these films have a wide range of potential applications in the field of food packaging to extend the shelf life of fresh produce.

## Introduction

Contamination and infection of pathogenic microorganisms pose a serious threat to public health (1–4). An investigation revealed that *Staphylococcus aureus* (*S. aureus*) was detected in 2.3% of fruits and vegetables in Sichuan Province of China (5). Consequently, there is a clear need to control the spread of food-borne pathogens and maintain the quality of food products (6). Historically, the food industry has reduced the effect of pathogen contamination by applying heat and by the addition of preservatives to inactivate undesired microorganisms (8, 9). However, thermal sterilization has some problems including inadequate inactivation of thermophilic bacteria and negative effects on the nutritional value of food (7). Preservatives may prolong the shelf life of food products to a degree, but the direct addition of preservatives in food usually compromises its antimicrobial activity and sometimes also produce negative effects on the organoleptic characteristics (10). Therefore, there is growing interest in developing active food packaging technologies, such as antibacterial absorbent pads, coatings, and films (11, 12). Various antimicrobial agents such as zinc ions, essential oils, chitosan, cinnamaldehyde, and quaternary ammonium salts have been extensively studied as the antibacterial components of active food packaging materials (13–17). However, the limitations of the significant and rapid antibacterial effect, as well as the lack of precisely control release of these agents, have brought major challenges to applying these films in the food industry.

**Figure 1:**
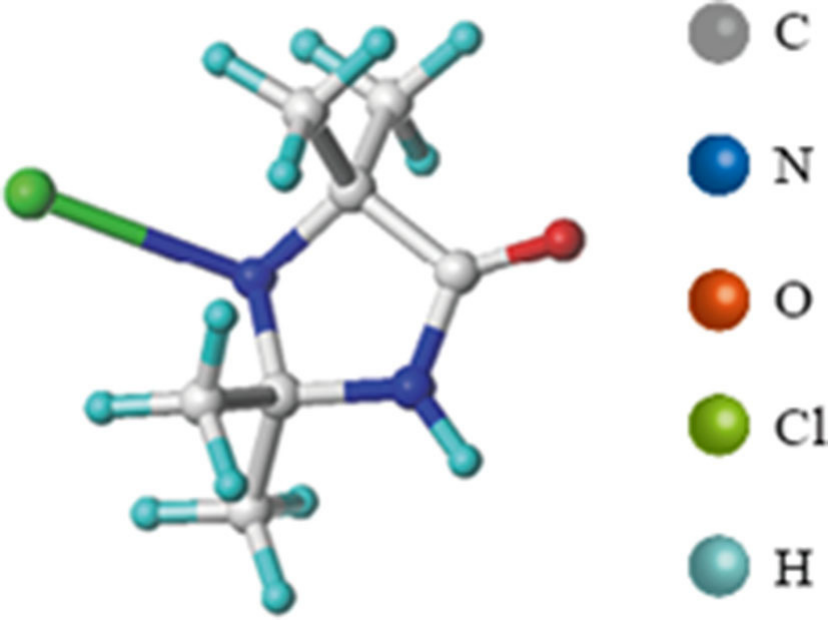
Structure of MC (1-chloro-2,2,5,5-tetramethyl-4-imidazoli-dinone).

N-halamines is a group of compounds containing one or more nitrogen-halogen covalent bonds, and many of these compounds have effective antibacterial activity (18, 19). Many N-halamines have been used in a variety of food contact surfaces to improve food safety by reducing cross-contaminations (20). 1-chloro-2,2,5,5-tetramethyl-4-imidazolidinone (MC, Figure 1) is a monochlorinated N-halamine, which presented a broad-spectrum antibacterial activity and long-term biocidal properties (21–23). Studies showed that polypropylene non-wovens coated with MC reduced 6 log (complete inactivation) of *S. aureus*, 4 log of *Escherichia coli* O157:H7 (*E. coli* O157:H7), and 7.5 log_10_EID_50_/mL of avian influenza virus within only minutes of contact, showing the promise as an application to respiratory masks and air filters (21, 24). In addition, Ren et al. incorporated MC into the absorbent pads of fresh beef packages, and results showed that the microbial load in the beef was significantly reduced and the shelf life of the beef was extended from 1 day to 11 days (25).

Discarded plastic materials often accumulate in the environment and is referred to as “white pollution.” Because of the severity of “white pollution” in the landscape and water all over the world, biodegradable materials of food packaging are in increasingly high demand (26, 27). Polylactic acids (PLA), obtained from lactic acid, is one of the most promising packaging materials. PLA not only has compostable properties, but also has comparable mechanical properties as polyethylene terephthalate (PET) and polystyrene (PS) (28). Multiple techniques including the addition of modifiers (29) have been applied for improving mechanical properties of PLA to improve its stiffness and brittleness.

In this work, we grafted MC onto PLA resins, in order to obtain an antimicrobial food packaging film with satisfactory mechanical strength and transparency. The film homogeneity and spatial distribution of added compounds were studied through scanning electronic microscopy (SEM) and Fourier transform infrared spectroscopy (FTIR). Mechanical strength, including tensile strength (TS) and elongation at break (EB) of films was also measured. In addition, their barrier properties of moisture and oxygen, water contact angle and thermal stability were investigated. The antibacterial activity of produced films against *E. coli* and *S. aureus* of the prepared films were evaluated through a “sandwich” test.

## Materials and Methods

### Materials

Polylactic acids (3052D) was supplied by Nature Works (Minnesota, MN). MC was supplied by Cangzhou Jincang Chemicals, LTD (China). Saline, sodium thiosulfate, and other chemicals were obtained from Xi'an Jingbo Biological Technology Co., Ltd. Luria–Bertani (LB) broth and LB agar were all purchased from Beijing Aoboxing Company. All the materials and reagents were used without further purification.

### Preparation of MC Incorporated PLA Film

Polylactic acids' powder was dissolved in chloroform at room temperature, sonication at 24°C for 60 min until fully dissolved. A pre-determined amount of MC (0, 0.05, 0.25, and 0.5 wt%) was added to the PLA solution, followed by sonication for 10 min to obtain a homogeneous solution. Then, the mixture was poured onto a stainless-steel plate, and then placed under a fume hood for 24 h to completely volatilize the solvent. Films were peeled from the plates and marked as the control, PLA–MC-0.05, PLA–MC-0.25, and PLA–MC-0.5, respectively.

### Antimicrobial Assays

Bactericidal experiments were performed on two typical foodborne bacteria, Gram-negative *E. coli* ATCC 25922 and Gram-positive *S. aureus* ATCC 25923. All the materials used in this study were sterilized by autoclaving for 20 min at 120°C. A typical colony from Luria–Bertani agar was selected and added in LB broth, incubating at 37°C for 24 h. Bacteria were collected by centrifuging at 10,000 rpm for 3 min and washed three times with 1.5 ml of saline, and finally re-suspended in 1 ml saline. An *E. coli* culture with an absorbance at 600 nm of 0.2 (about 6 × 10^7^ CFU/ml) and *S. aureus* with an absorbance at 600 nm of 0.4 (about 1.3 × 10^7^ CFU/ml) were used for the following experiments.

Briefly, an aliquot of 25 μl model bacterial suspension was inoculated onto the center of a 2.5 × 2.5 cm^2^ sample and covered with another identical one, and then 18 g of sterile weights were placed onto the “sandwich” to ensure full contact of the test samples with the bacteria. After a pre-determined period of contact time (5, 10, and 30 min), the swatches were treated with 5 ml of 0.03% Na_2_S_2_O_3_ solution to quench the oxidative chlorine of the samples without affecting the microbial growth. Then, the mixtures were vortexed for 2 min to transfer the adhered microbial cells into the suspension. Ten-fold serial dilutions of the resulting suspension were conducted with saline, and 100 μl of each dilution was placed onto the LB agar plates, followed by incubation at 37°C for 24 h for the bacteria. The colony forming unit (CFU) on each plate was recorded for antimicrobial efficacy analysis.

### Film Characterizations

#### Morphology Characterization

The surface and boundary morphologies of the films were observed by scanning electron microscope (Hitachi Regulus 8100, Japan). Samples were sputter-coated with gold for 60 s before SEM analysis and images were observed under high-vacuum mode at an operating voltage of 60 kV. The image scales were set at 1:1,000 and 1:6,000, respectively.

The FTIR spectra of the PLA films and PLA–MC films were obtained between wavenumbers 400 and 4,000 cm^−1^ using a FTIR spectrometer (Perkin Elmer Corporation, USA). Spectra were recorded from 30 to 1,000 cm^−1^ with the resolution of 2 cm^−1^. The absorption bands of the main and additional functional groups were identified and observed for any optical changes (30).

#### Mechanical Properties

Mechanical properties tests including tensile strength (TS) and elongation at break (EB) were conducted under ambient temperature by a texture analyzer (TA. XT. plus, Stable Micro System) and calculated following the method published by Zhang's et al. (31). The films were cut into 1 × 6 cm strips perpendicularly to the direction of flow, and film samples were placed in a relative humidity (RH) chamber at 75% for 24 h. Prior to testing, the film thickness was measured at 5 randomly selected points on each sample using a digital micrometer (Sato Ding, Osaka, Japan). The thickness value is the average value of 5 measurements. The testing conditions used were cross head speed of 5 mm/min and fixed at an initial grip distance of 40 mm. TS and EB were calculated according to the following formula:


(1) $$\begin{array}{lll} \text{TS}\left(\text{MPa}\right) & = & \frac{Force\;at\;break\;\left(N\right)}{Thickness\left(mm\right)\times width\left(mm\right)} \end{array}$$

(2) $$\begin{array}{lll} \text{EB}\left(\text{\%}\right) & = & \frac{Elongated\;length\left(mm\right)}{Original\;length\;\left(mm\right)} \end{array}$$

### Barrier Properties

#### Oxygen Permeation Property

The oxygen transmission rate (OTR) was determined based on the differential pressure method using a VAC–VBS model oxygen permeation analyzer (Labthink Instruments Co., Ltd., Jinan, China). Tests were performed with circular films clamped in a diffusion chamber and all measurements were performed in triplicate. Oxygen was introduced to the upper half of the diffusion chamber, and the lower half was under vacuum. To better compare the results with other reported work, the values were expressed in the commonly used unit of cm^3^·m^−2^·24 h·0.1 MPa.

#### Water Vapor Permeation Property

The steady-state water vapor permeation (WVP) of the film samples were measured according to the Chen's et al. group method with some modifications (32). Film samples were covered on the top of centrifuge tubes containing about 41 g of dry anhydrous silica gel, then moved to a desiccator containing saturated sodium chloride solution (75% RH). The tubes were weighed after 24 h to record the amount of transferred water. The WVP of the film was calculated as the following equation:


(3) $$\text{WVP}=\frac{q}{t}\frac{d}{s\Delta P}$$

where: water vapor transmittance (WVP), (g·m·m^−2^·Pa^−1^·s^−1^); q/t—increased weight of moisture permeable tube in unit time, g/s; d—sample film thickness, m; s—test area of sample film, m^2^; ΔP—vapor pressure on both sides of the sample, Pa.

#### Water Contact Angle

The water contact angles (WCAs) of native and modified films were measured using the Theta Optical Tensiometer (Attention, OCA 20, Dataphysics, Germany). The static contact angle was measured using deionized water dropped by a micropipette within 10 s. The volume of the water droplet was 10 μl. The WCA was measured by the tangent method with an accuracy of 0.1°. Water droplet images were collected and imported into image analysis software by five points fitting method to evaluate the moisture absorption of water droplets. WCA measurements were made at five different locations on the film and the average values were reported.

### Thermal Stability

The thermal stability was performed on a thermogravimetric analysis instrument (TA TGA5500, USA). Thermal analysis was conducted to investigate the influence of the addition of active compound on the thermal stability of the PLA film. Approximately 5 mg of film samples were heated from 30 to 600°C at a heating rate of 10°C/min under a nitrogen atmosphere.

### Packaging of Fresh Produce

Fresh strawberries from local market were set in PE trays without washing, covered with produced packaging films mentioned above, and sealed with parafilm. Control groups were strawberries with PLA film developed in the previous section, packaging and storing at the same condition with the treatment group. All the products were stored at 25°C, 50% RH to mimic the local storage conditions of the commercial products. Observations were conducted twice a day to examine any forms of decay.

### Statistical Analysis

GraphPad (version 6.0, Prism, San Diego, CA) was used to analyze data and generate graphs of the results. The differences between treatments were investigated by one-way ANOVA and followed by a Tukey's multiple comparison test. Statistical analyses were conducted at the 95% confidence level.

## Results and Discussion

### Characterization of Films

#### Morphology and Structure

**Figure 2:**
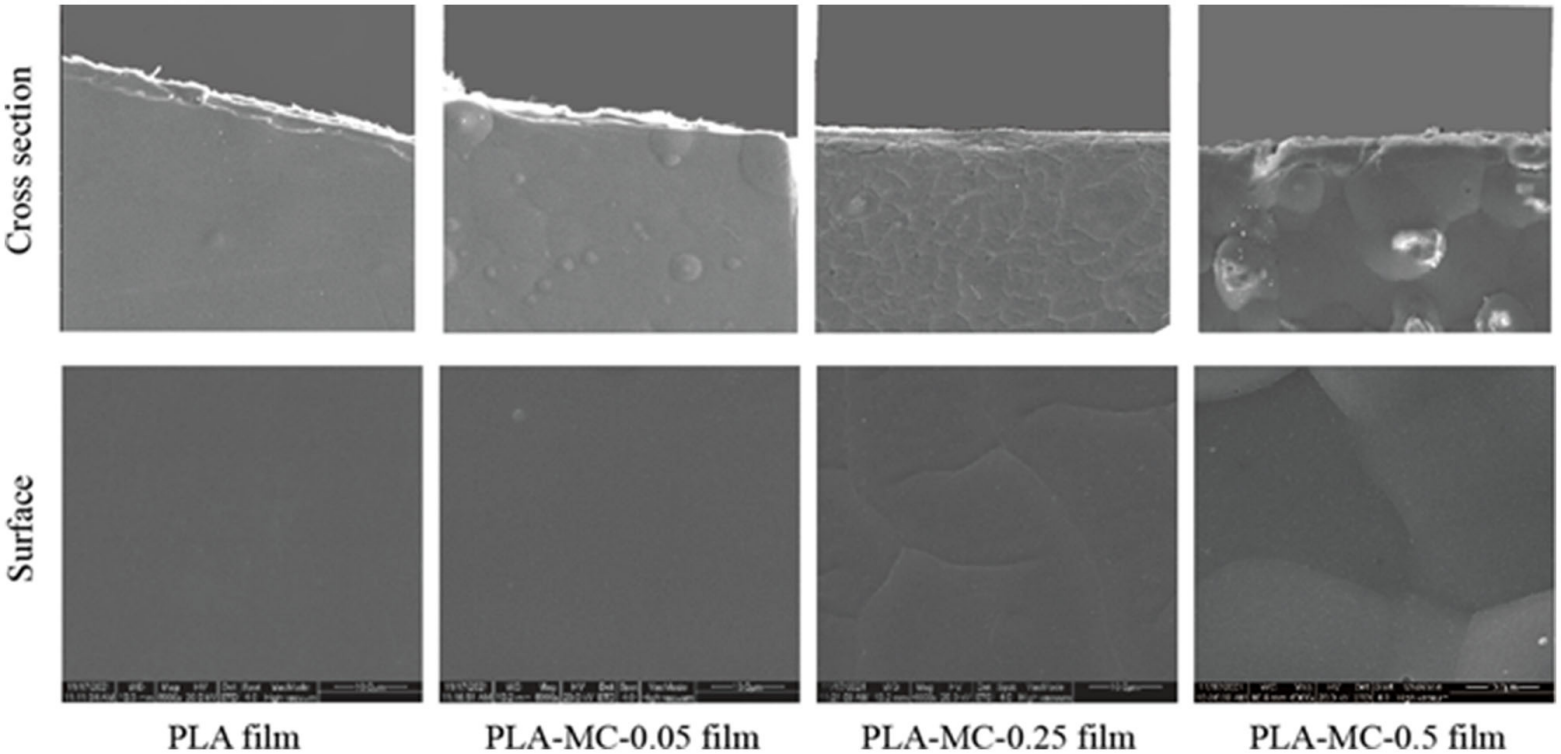
Surfaces (lower row) and cross sections (upper row) of the PLA and PLA–MC films under scanning electron microscopy (SEM), scale bar = 10 μm.

Scanning electronic microscopy images of the surfaces and cross-sections of the active PLA–MC films are presented in Figure 2. The surface morphology of the film was controlled by the amount of MC in the polymer solution. As demonstrated in Figure 2, the surfaces of all the original PLA and active PLA–MC films were smooth, compact, and homogeneous, except for PLA–MC-0.5. PLA film incorporated with 0.25% MC showed a compact cross-section. It appears that appropriate amounts of MC in the polymer acted as a plasticizer and impacted the interactions between PLA molecular. High loadings of MC caused self-aggregation and interrupted the continuous interactions between PLA molecular chains, leading to an uneven surface and rough section with many bubbles.

**Figure 3:**
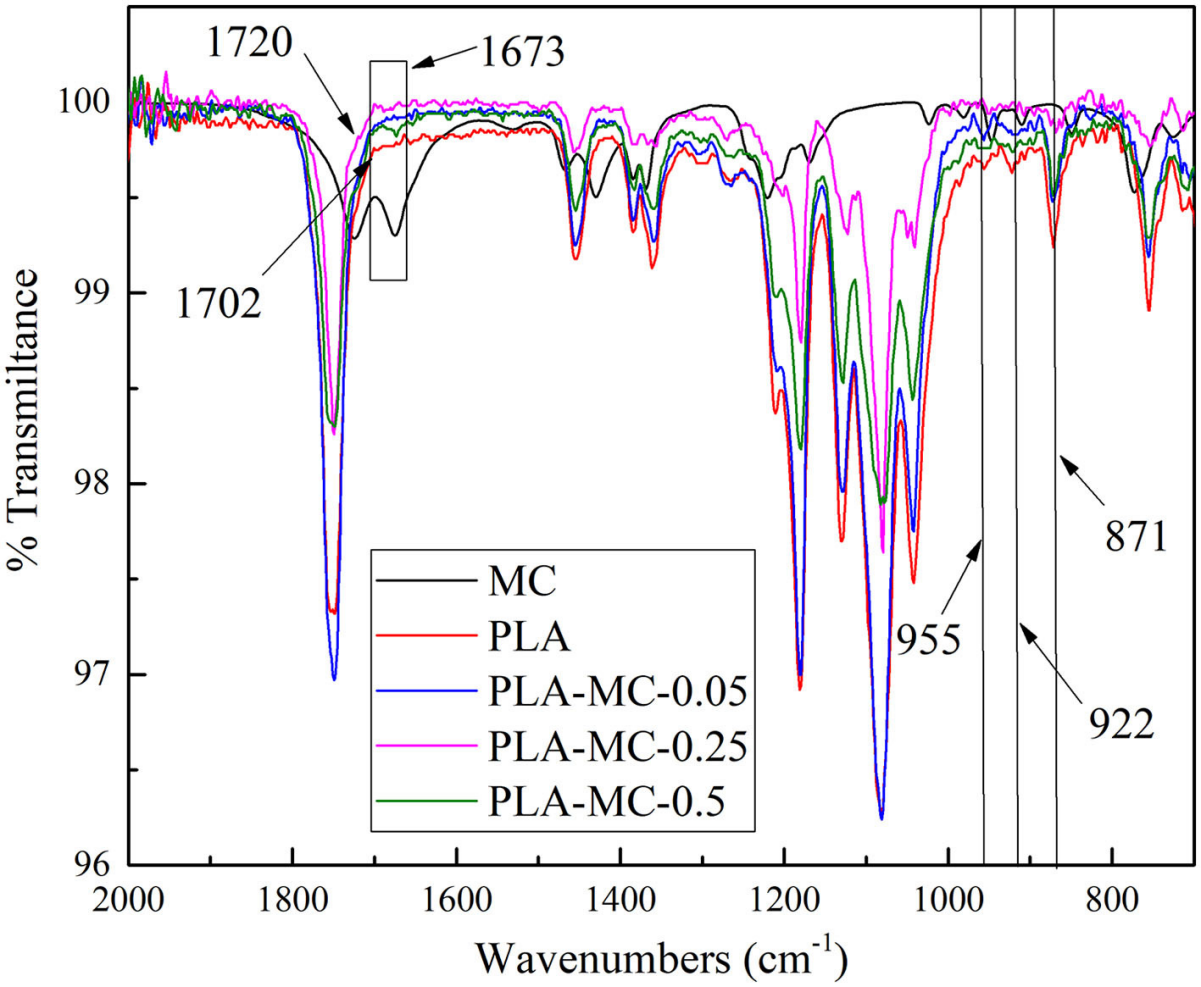
FT–IR spectra of PLA film, PLA–MC-0.05 films, PLA–MC-0.25 and PLA–MC-0.5 films: magnification of the region of 2,000–700 cm^−1^.

Supplementary Figure 1 illustrates the synthesis procedures of MC incorporated PLA films. MC contains methyl radical that can react with PLA *via* free radical substitution. The FT–IR spectra of PLA and PLA–MC are shown in Figure 3. It presents typical bands of PLA and functional group interactions on the film surface in terms of absorption peaks in specific regions (32). As reported in the previous studies, several typical vibration bands of the PLA film, including C = O stretching (1,746 cm^−1^), CH3 bending (1,465 cm^−1^), C–O–C stretching (1,181 cm^−1^), C–O stretching (1,127 and 1,083 cm^−1^), and OH bending (1,014 cm^−1^) are detected in the FTIR spectra (33–35). The presence of new peak at 1,673 cm^−1^ on PLA–MC was assigned to the stretching vibration of planar imidazolidinone ring (36). The vibrational band shifted from 1,702 to 1,716 cm^−1^ as a result of the electron-withdrawing effect of the oxidative chlorine (37). Thus, FTIR spectra provided the evidence of the successful incorporation of MC onto the films. The graft polymerization procedures did not mask the active nitrogen–chlorine bond moieties in MC; thus, the films would be expected to retain the bactericidal properties of MC.

In addition, at lower wavenumbers three vibrational peaks appeared at 955, 922, and 871 cm^−1^, that are attributed to the crystallinity of PLA (38). The peaks at 922 and 871 cm^−1^ were related to the α-crystals of the semicrystalline PLA, while the peak at 955 cm^−1^ reflected the amorphous part of the polymer. Thus, the reductions of the 922 and 871 cm^−1^ peaks, along with the simultaneous decrease of the 955 cm^−1^ peak when MC raised, indicated that the presence of MC served as a filler to prevent the crystallization of PLA.

#### Physical Properties

**Figure 4:**
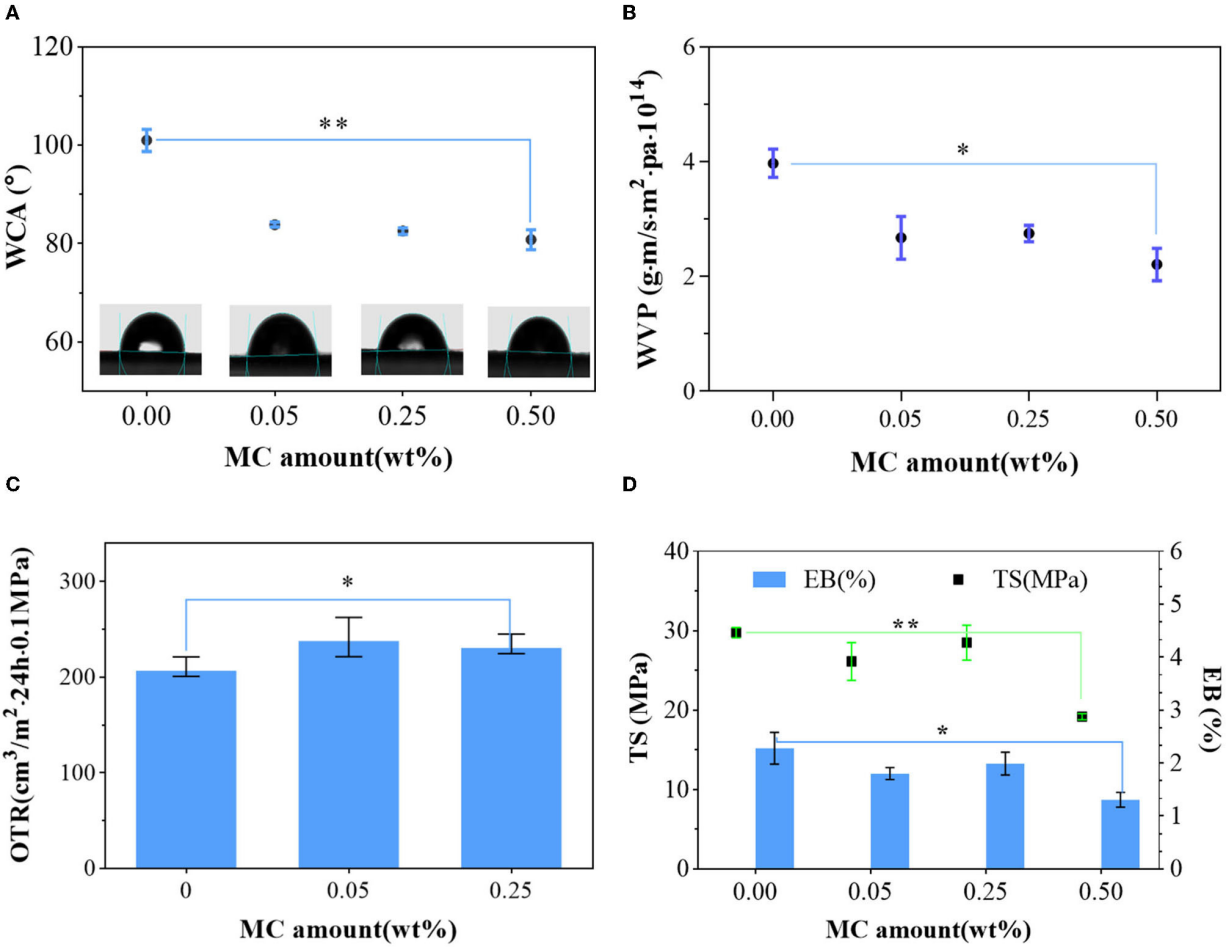
Mechanical and barrier properties of the PLA film and PLA–MC films: **(A)** microscopic images of water droplets on the surface of PLA film and different PLA–MC films, and the corresponding water contact angle (WCA) values; **(B)** water vapor permeability (WVP) of PLA film and PLA–MC films; **(C)** oxygen transmission rates (OTRs) of PLA film and PLA–MC films; **(D)** tensile strength (TS) and elongation at break (EB) between PLA film and PLA–MC films. *indicates the difference is significant at the 0.05 level. **indicates the difference is significant at the 0.01 level.

Figure 4A shows the hydrophilicity of the original PLA and the modified film. Many ester bonds on the PLA film led to a contact angle of 101°, which was categorized as a hydrophobic material (θ > 90°). After N-halamine modification, the hydrophilicity of films was improved with a water contact angle (WCA) of 80.8°. The improvement of hydrophilicity is beneficial for ensuring a good contact between films and microorganisms which effectively enhances the bactericidal property of the film. Increased hydrophilicity impaired the resistance to water, which is consistent with the results of WVP. In addition, the hydrophobicity of PLA–MC film was higher than that of many reported polymer films, such as polyprotein (71.4°), carrageenan film (63.8°), and gelatin (57.2°), which still had the durability required for a food packaging materials (39–41).

The WVP of PLA film was 3.98 × 10^−14^g·m·m^−2^·Pa^−1^·s^−1^, whereas the WVP of PLA–MC films were reduced generally with the addition of MC (Figure 4B). The decrease of WVP was due to the dense structure from molecular interactions of films with MC, which consequently blocked the route of moisture migration (Figure 2). The WVP values of the active PLA films with 0.25 wt% MC appeared to be slightly higher (2.75 × 10^−14^g·m·m^−2^·Pa^−1^·s^−1^) than those of films with 0.05 wt% MC (2.68 × 10^−14^g·m·m^−2^·Pa^−1^·s^−1^), which we assign to the polar nature of MC. These values are superior compared to many packaging biopolymers, such as gelatin (~0.84 × 10^−9^g·m·m^−2^·Pa^−1^·s^−1^), chitosan (~1.26 × 10^−9^g·m·m^−2^·Pa^−1^·s^−1^), and thermoplastic starch (~2.9 × 10^−10^g·m·m^−2^·Pa^−1^·s^−1^) (40, 42, 43). These data show that PLA–MC films can provide a strong water vapor barrier, thereby reducing the impact of moisture migration on the packaged food.

As shown in Figure 4C, the PLA film has an OTR of 210.999 cm^3^·m^−2^·24 h·0.1MPa at 20°C, 25% RH. Film at 0.5% MC were not able to be tested through the OTR because of its inadequate mechanical properties. Upon the dispersion of MC, the OTR of PLA–MC films rose, since most free polar MC was interacted with PLA molecules and made it easy for oxygen to pass through the film. When the amount of MC increased, the OTR dropped slight due to the dense and organized structure of the polymer matrix. The change in the oxygen barrier capacity of the PLA–MC film was consistent with the trend of WVP. As an excellent water barrier and good oxygen barrier properties were usually not compatible, and materials with enhanced hydrophobicity tend to exhibit poorer oxygen barrier capacity (39). Nevertheless, the OTR of the PLA–MC-0.25 films (234.85 cm^3^·m^−2^·24 h·0.1MPa) is still lower than many reported biopolymer films, such as the ethylene–vinyl alcohol copolymer (EVOH) film (9,500.16 cm^3^·m^−2^·24 h·0.1MPa), polylactic acids–polyhydroxybutyrate (PLA–PHB) film (513.9 cm^3^·m^−2^·24 h·0.1MPa) and polylactic acid–poly (butylene succinate adipate) (PLA–PBSA) film (266.39 cm^3^·m^−2^·24 h·0.1MPa) (28, 44, 45).

Proper mechanical performances provide adequate assurance for packaging materials to prevent breakage during transportation and storage. Maintaining prominent mechanical properties of antibacterial materials during application is also highly desired. Supplementary Figure 2 shows a large PLA–MC piece in a size of 7 × 14 cm. Taking the unique flexibility performance of the PLA, the film tolerated large bending, folding, rolling, and twisting deformations without any fracture, as demonstrated in Figure 4 (46). PLA and PLA–MC films were homogeneous without any visual cracks, scratches, bubbles, or phase separation. Samples thickness was constant about 0.09–0.10 mm with no significant differences (*p* < 0.05) with the incorporation of MC. Figure 4D shows the typical tensile stress–strain diagrams of pure PLA films and PLA films with MC. TS and EB values varied in range of 19.17–29.79 MPa and 1.31–2.28%, respectively. The synthesized PLA film exhibited the highest breaking strength and elongation. However, with more MC introduced, the mechanical performances of the resulting copolymer films exhibited a significant fluctuation (*p* < 0.5). PLA–MC film, with 0.25 wt% MC exhibited the strongest breaking tensile stress of 28.5 MPa and a strain of 1.99%. Therefore, on the premise of ensuring good antibacterial performance, considering the influence of MC content on TS and EB, samples with mass ratio of 0.25 wt% MC would be recommended for further research. In addition, the TS value in this study was even higher than that of similar antibacterial films containing chitosan (17.99 MPa) (47). Therefore, PLA–MC-0.25 film is a tough and durable material that shows promise for use in food packaging for various products.

#### Thermogravimetric Analysis

The thermal stability and the effect of the adding MC on the stability of PLA membranes were determined with TGA. Supplementary Figure 3 represents the TGA thermal analysis diagram of PLA and PLA–MC films. The TGA curves of PLA treated with or without MC exhibited a similar weight loss tendency. The degradation of film was observed in two stages: the first occurred over a temperature below 320°C, mainly attributed to the loss of moisture. The second loss occurred over a temperature ranged from 320 to 450°C, ascribed to the thermal degradation of PLA. A new peak appeared near 127°C, which could be attributed to the decomposition of grafted MC structure (PLA–MC), having been proven by FT–IR results. Due to the existence of MC, the weight loss is reduced, and the remaining material at temperatures lower than 400°C decreases with MC content. Overall, PLA–MC film showed a good thermal stability.

#### Antibacterial Activity

**Figure 5:**
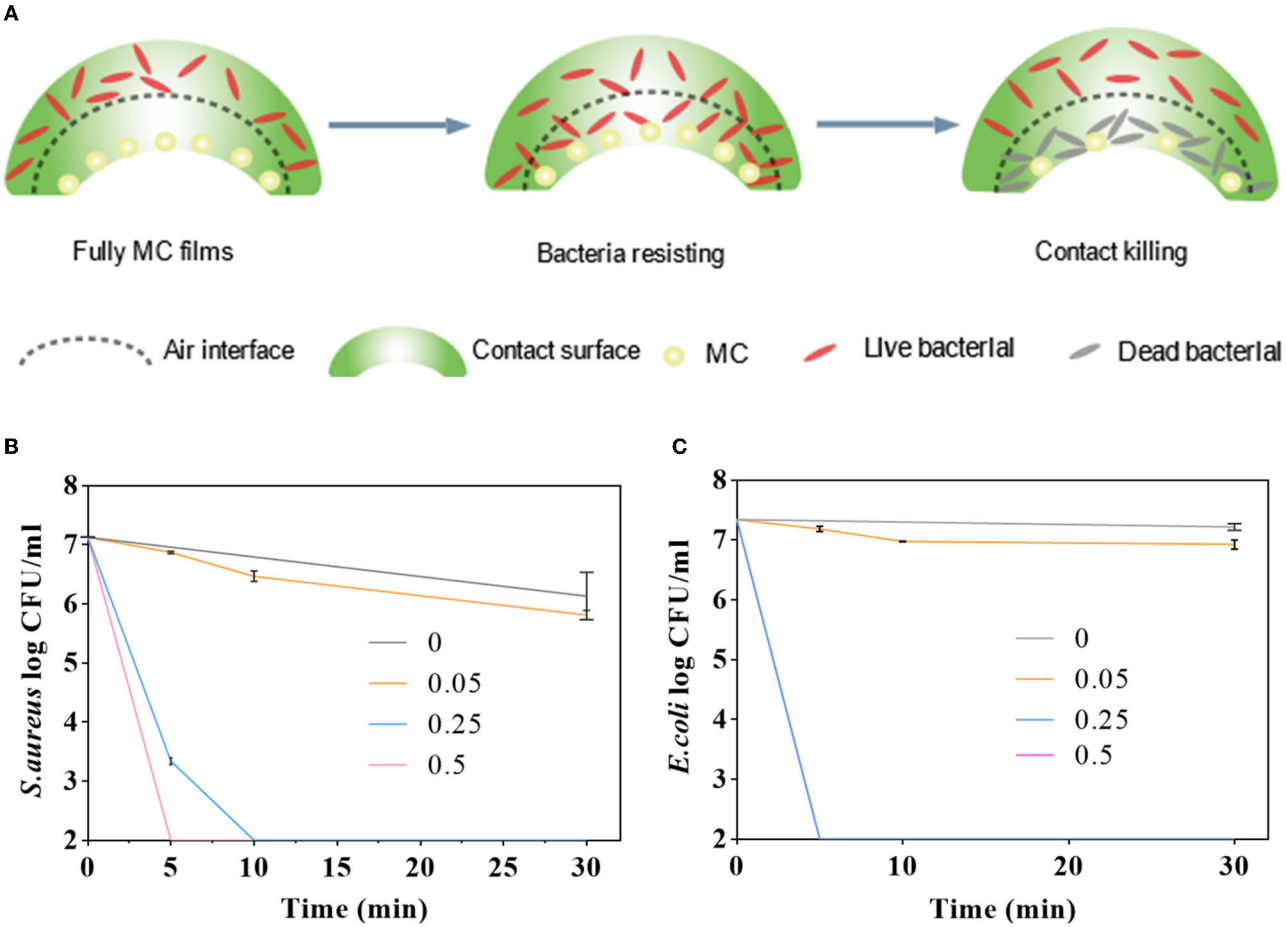
**(A)** Diagrammatic sketch of antibacterial mechanism of the as-synthesized MC-containing polylactic acid films; antimicrobial activities of the control films (PLA film), PLA–MC-0.05, PLA–MC-0.25, and PLA–MC-0.5 films against **(B)** *S. aureus* and **(C)** *E. coli*, respectively.

To evaluate the antimicrobial activity of the PLA–MC films, the surface was challenged with *E. coli* or *S. aureus*, and the microbial loads were assessed by 10-fold serial dilution, plating, and incubation. Figures 5B,C shows the antibacterial activities of PLA and PLA–MC films against *E. coli* and *S. aureus in vitro*. Figure 5A depicts the biocidal function pathways of the PLA–MC. Where the bacteria comes into contact with the packaging film, the MC groups release oxidizing chlorine that disrupts the membrane cell of the bacteria (48). When the pathogens come into contact with the PLA–MC films, the active chlorine of the amide-halamine located on the external surface could be consumed first *via* a direct contact killing pathway, converting to N-H structures. Then, the active chlorine transferred from the internally chlorinated hydantoin to such amide structures, ensuring the durable high biocidal activity of the PLA–MC surface (46).

As shown in Figure 5B, nearly no contact-killing was observed on control film. In contrast, the PLA–MC film showed rapid and effective reductions in the amount of *S. aureus*. In the case of PLA–MC-0.5, the film displayed 7 logs of CFU reduction in merely 5 min contact, which was corresponding to a promising contact-killing efficacy of 100%. The PLA–MC-0.25 still achieved 7 logs reduction in 10 min of contact, respectively. It is of interest that the PLA–MC-0.05 sample also did not provide an antimicrobial property. This was probably due to the insufficient contact of microorganisms with the MC, which is sparsely distributed in the film. Because the mechanism of antimicrobial action of N-halamines is the direct transfer of oxidative halogen to microbial cells, spaces among the MC of PLA–MC-0.05 on the film surface had no direct contact of the polymer with the bacteria, resulting in undetectable biocidal action. Figure 5B exhibit a time-dependent antimicrobial effect of the PLA–MC films. The control films did not provide any noticeable reduction of bacteria even after 30 min of contact. In contrast, both PLA–MC-0.25 and PLA–MC-0.5 films showed 7 logs reduction of *S. aureus* within 30 min of contact. In addition, the PLA–MC-0.5 film achieved 7 logs reduction in 5 min of contact for *S. aureus*, which was nearly 6 times faster than that of the PLA–MC-0.25 film. As discussed earlier, the added MC structure increased the hydrophilicity of the films with reduced WCA of 10.7°, and the improved hydrophilicity of the films ensured better contact with bacteria, resulting in faster biocidal functions of the films.

As shown in the Figure 5C, the antibacterial effect on *E. coli* depends on both the added amount of MC and time of contact. PLA–MC-0.05 film had no obvious antibacterial effect when the content of MC was less. Other PLA–MC films produced 7 logs bactericidal effect on *E. coli* within 30 min. The difference is, the PLA–MC-0.25 inactivated 7 logs *E. coli* within 5 min of contact, and the swatches inactivated *S. aureus* <2 logs within 5 min of contact time. The antimicrobial efficacy for *E. coli* were much higher than the *S. aureus* group. The different inactivating rates for these two bacteria might due to the difference of cell wall structures, which the Gram-positive bacteria have thicker layers of peptidoglycan resulting in the slower penetration of MC into bacterial cells (49).

#### Preservation Ability

**Figure 6:**
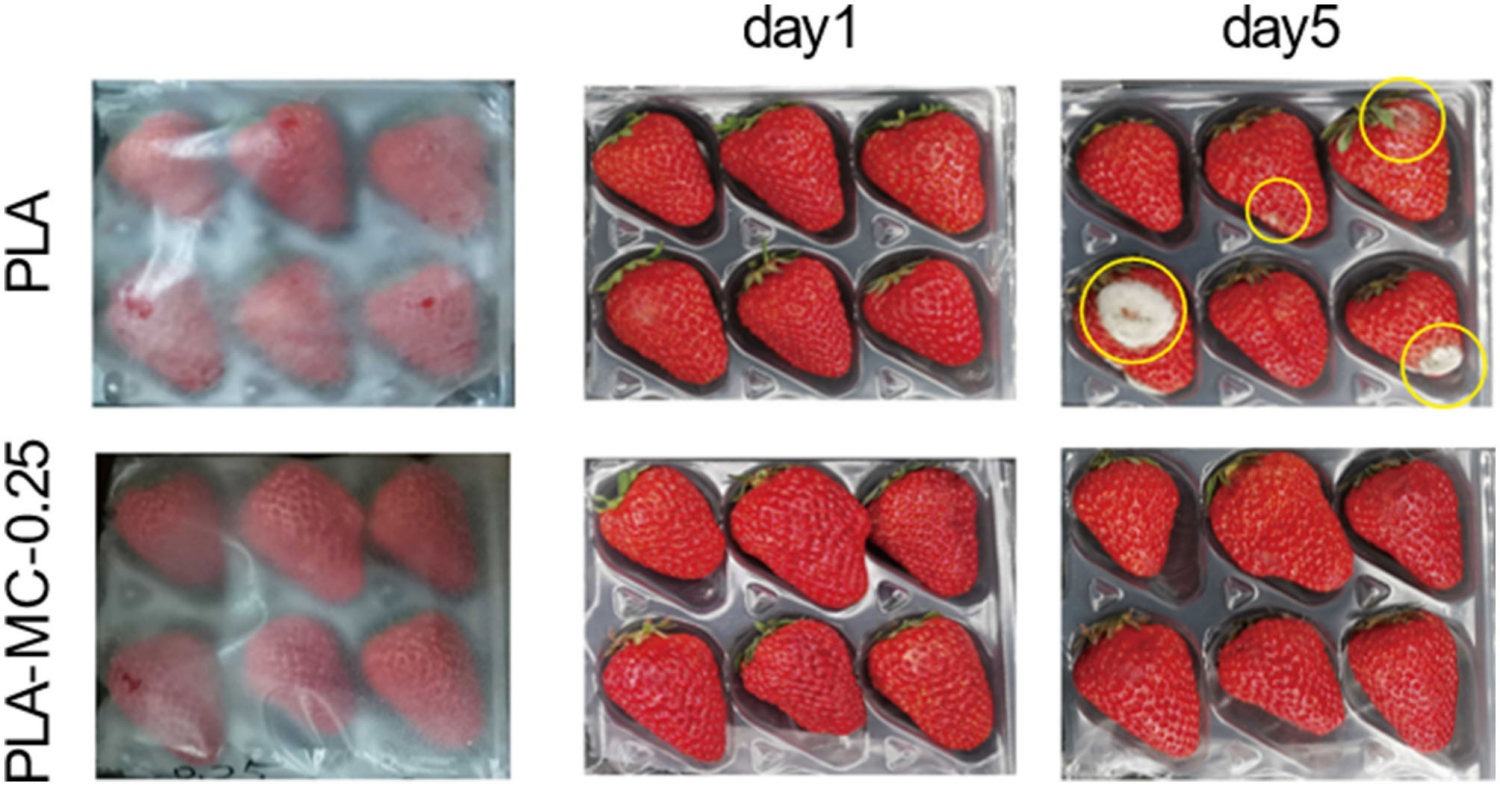
Strawberries packaged with the PLA films and the MC–PLA-0.25 on days 1, 3, and 5.

We designed a straightforward strawberry storage experiment to assess food preservation efficiency of the PLA–MC films. After 5 days of storage, decays including white molds and dark spots were present apparently on the strawberries of the control groups, and exudates were observed from some of the spoiled strawberries (Figure 6). While most strawberries covered with the PLA–MC-0.25 film still exhibited an appealing appearance as the fresh ones. Decays occurred mainly because of the microbial growth. MC incorporated films showed effective antimicrobial activity, and thus maintaining the quality of packaged strawberries. This study implied that the PLA–MC films are promising packaging materials to extend the shelf life of fresh produce.

## Conclusion

This study produced an active food packaging film with highly mechanical resistant PLA films and antibacterial compound MC. N-halamine MC has been successfully combined onto the PLA *via* substitution reaction. Characterizations of both SEM of PLA and PLA–MC films showed that the MC dispersed uniformly in PLA films. PLA–MC films had a high transparency, strong mechanical strength, thermal stability, water vapor barrier, and oxygen permeability property. The PLA–MC-0.25 films inactivated 7 logs (complete inactivation) of *S. aureus* and *E. coli* within 30 and 5 min of contact, respectively. In the pilot study, strawberries wrapped with MC incorporated films extended their shelf life to at least 5 days at room temperature. The findings in this work indicate that PLA with 0.25% MC can be exploited as packaging materials of fresh produce to extend their shelf life.

## Data Availability Statement

The original contributions presented in the study are included in the article/Supplementary Material, further inquiries can be directed to the corresponding author.

## Author Contributions

LA: methodology and writing—original draft preparation. XH: software. PP: writing—review and editing. TR: supervision and project administration. All authors contributed to the article and approved the submitted version.

## Funding

The authors would acknowledge the research funds from the National Natural Science Foundation of China (32001630) and Shaanxi Key Research and Development Program (2020NY-151).

## Conflict of Interest

PP was employed by Solaster LLC. The remaining authors declare that the research was conducted in the absence of any commercial or financial relationships that could be construed as a potential conflict of interest.

## Publisher's Note

All claims expressed in this article are solely those of the authors and do not necessarily represent those of their affiliated organizations, or those of the publisher, the editors and the reviewers. Any product that may be evaluated in this article, or claim that may be made by its manufacturer, is not guaranteed or endorsed by the publisher.
