# Supplementary material for: A Sustainable and Antimicrobial Food Packaging Film for Potential Application in Fresh Produce Packaging
Source: Front Nutr. 2022 Jul 7;9:924304. doi: 10.3389/fnut.2022.924304 (PMC9301339; doi:10.3389/fnut.2022.924304)
Supplement: Supplementary file 1 [file Presentation_1.pdf]

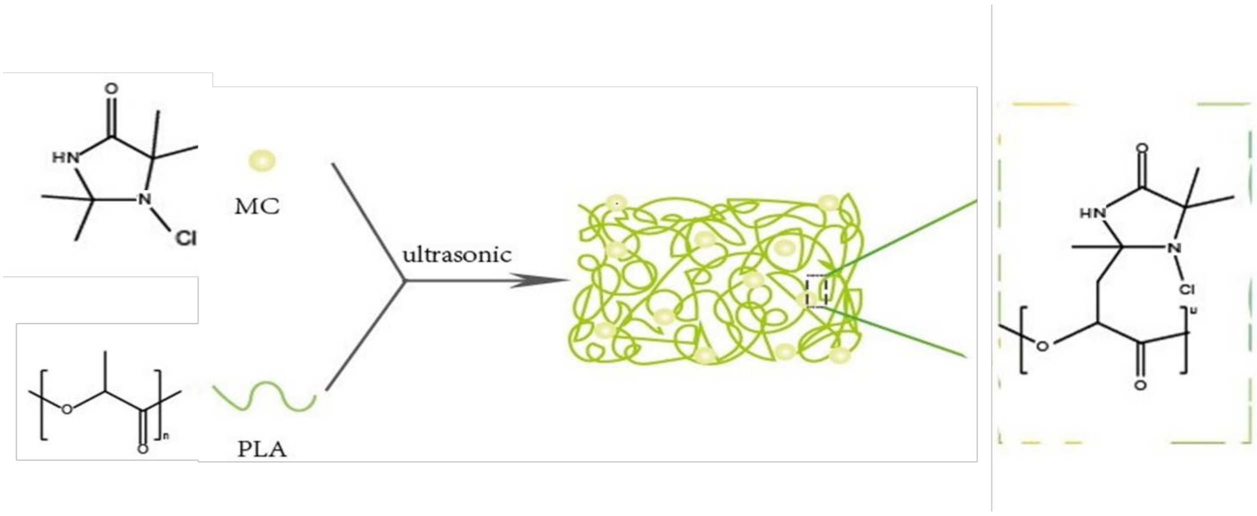

FIGURE S1| Schematic illustration of N-halamine-containing PLA films.

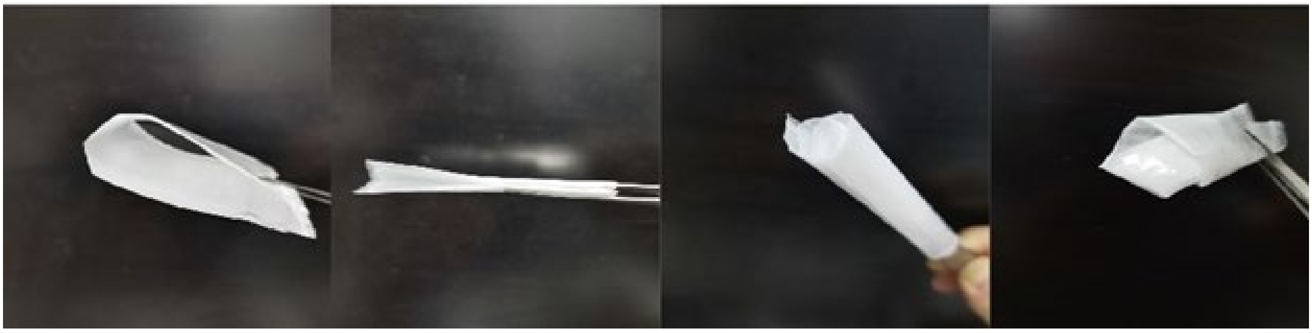

FIGURE S2| optical images of an PLA-MC-0.05 film after bending, folding, rolling, and twisting.

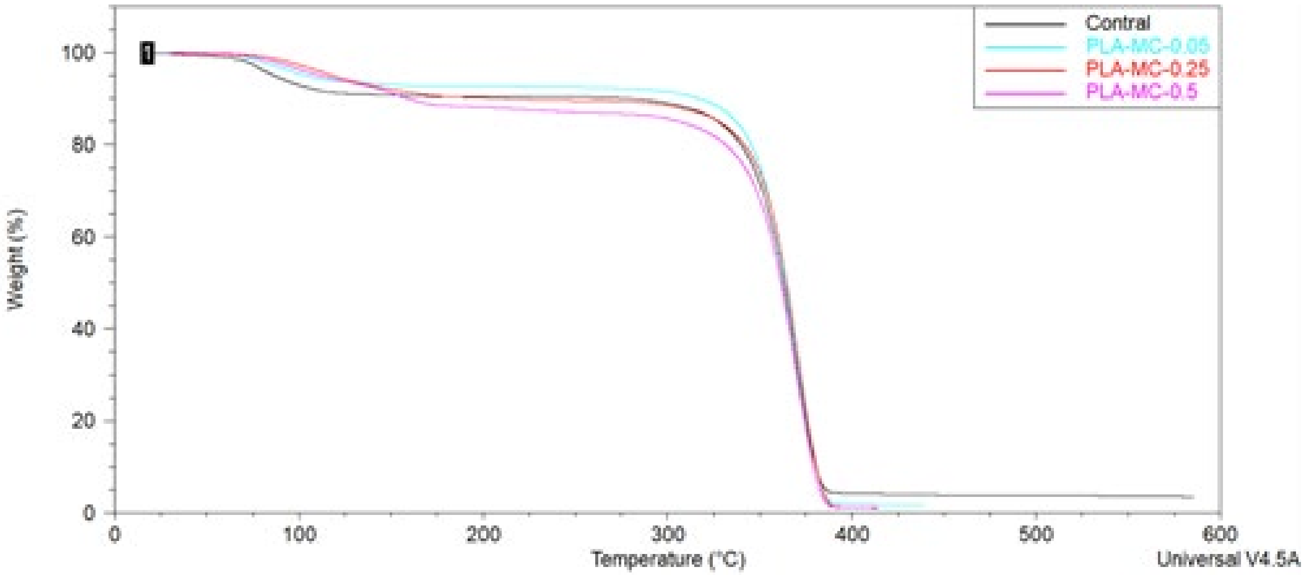

FIGURE S3| Thermo gravimetric analysis (TGA) curves of PLA film and PLA-MC films.
